# Vitamin A/Retinol and Maintenance of Pluripotency of Stem Cells

**DOI:** 10.3390/nu6031209

**Published:** 2014-03-21

**Authors:** Jaspal S. Khillan

**Affiliations:** Department of Immunology, University of Pittsburgh, 3501 Fifth Ave, Pittsburgh, PA 15261, USA; E-Mail: khillan@pitt.edu; Tel.: +1-412-383-6987; Fax: +1-412-648-8695

**Keywords:** vitamin A, retinol, retinoic acid, embryonic stem cells, cancer stem cells, PI3 kinase signaling, OCT4, NANOG, SOX2, Insulin like growth factor 1 receptor (IGF1R)

## Abstract

Retinol, the alcohol form of vitamin A is a key dietary component that plays a critical role in vertebrate development, cell differentiation, reproduction, vision and immune system. Natural and synthetic analogs of retinol, called retinoids, have generally been associated with the cell differentiation via retinoic acid which is the most potent metabolite of retinol. However, a direct function of retinol has not been fully investigated. New evidence has now emerged that retinol supports the self-renewal of stem cells including embryonic stem cells (ESCs), germ line stem cells (GSCs) and cancer stem cells (CSCs) by activating the endogenous machinery for self-renewal by a retinoic acid independent mechanism. The studies have also revealed that stem cells do not contain enzymes that are responsible for metabolizing retinol into retinoic acid. This new function of retinol may have important implications for stem cell biology which can be exploited for quantitative production of pure population of pluripotent stem cells for regenerative medicine as well as clinical applications for cancer therapeutics.

## 1. Introduction

Vitamin A, a small dietary component of 286 Da size, discovered almost 100 years ago [1,2], is essential for vertebrate embryogenesis, normal growth and development [3,4,5,6] and its deficiency leads to reproductive failure in females [7]. It is a fat-soluble vitamin that belongs to the family of compounds which contains retinol and β-carotenes.

Vertebrates are unable to synthesize vitamin A which is generally acquired from carotenoids present in plants and through the food items absorbed from small intestine [8,9]. β-Carotene, also known as pro-vitamin A, is converted into vitamin A when additional levels are required.

Retinol is the alcohol form of vitamin A. Studies of the past two decades have shown that retinol is associated with cell differentiation via its most potent metabolite retinoic acid [10]. Although the physiology of its metabolites retinaldehyde [11] and retinoic acid is studied in well details, a direct biological function of retinol itself has not been fully elucidated. Recent studies however, have provided new evidence that retinol has a direct retinoic acid independent function in stem cell biology [12,13,14,15,16] and metabolic fitness of mitochondria [17]. In this chapter, I will review studies on the direct retinoic acid independent function of vitamin A/retinol in stem cell biology and its potential applications in the regenerative medicine. For retinoic acid mediated function of vitamin A/retinol, readers are advised to refer to several elegant reviews by other authors [18,19,20]. I anticipate that this review will provoke new ideas and bring into sharp focus further studies on the exploration of vitamin A/retinol function in the cell biology.

## 2. Retinol, Retinoic Acid and Cell Differentiation

Vitamin A/retinol is generally stored in the liver as retinyl esters primarily in the form of retinyl palmitate [21]. The stored vitamin A/retinol is mobilized into blood plasma by the enzymes retinyl ester hydrolases (REHs) [22]. The normal concentration of retinol in blood plasma varies between 1.0 and 2.0 μM [23].

**Figure 1:**
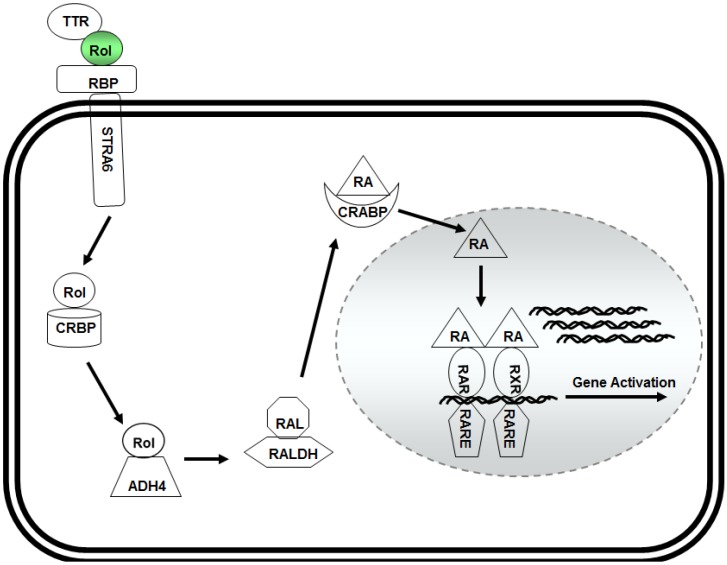
Model for retinol metabolism in a target cell: vitamin A/retinol (Rol) binds with the transthyretin (TTR) and retinol binding protein (RBP) to form a ternary complex that binds to STRA6 receptor on the surface of the target cell. Inside the cytoplasm, retinol binds to cellular retinol binding protein (CRBP) and is oxidized to retinaldehyde (RAL) by alcohol dehydrogenase (ADH4). RAL is then converted into retinoic acid (RA) by retinaldehyde dehydrogenases (RALDH). Retinoic acid isthen transported into the nucleus by binding to the cellular retinoic acid binding receptor (CRABP) where it forms a complex with RAR and RXR receptors that binds with retinoic acid response elements (RARE) in the promoter region of responsive genes to activate their expression.

The past two decades of research investigations have established that vitamin A/retinol executes its function via retinoic acid (Figure 1), and regulates the function of >500 genes involved in development and cell differentiation [24]. The circulating retinol in blood plasma binds to a 21 kDa retinol binding protein (RBP) also known as RBP4 and thyroxine binding-protein transthyretin (TTR) to form a ternary retinol-RBP-TTR complex in 1:1:1 molar proportion that binds to the target cell via cell surface receptor STRA6 (stimulated by retinoic acid 6) [25] for transport into the target cell.

Inside the cytoplasm, vitamin A/retinol binds to a 15 kDa cellular retinol binding protein (CRBP) and is converted into retinoic acid by two sequential oxidation steps that convert first retinol into retinaldehyde and then to retinoic acid. Though the conversion of retinol into retinaldehyde is reversible, the retinoic acid cannot be reduced back to retinol. Retinol is converted into retinaldehyde by retinol dehydrogenases (Rdh10) whereas the enzymes that metabolize retinaldehyde into retinoic acid include retinaldehyde dehydrogenases Ralhd1 (Aldhd1), Ralhd2 (Alhd1A2) and Ralhd3 (Alhd1A3) [26,27].

Retinoic acid is then transported to the nucleus where it binds to heterodimer receptors, retinoic acid receptor (RAR) and retinoid X receptor (RXR) which belong to the superfamily of ligand-inducible transcriptional regulators that include steroid hormone receptors, thyroid hormone receptors, and vitamin D3 receptors. This complex then binds to the retinoic acid responsive elements (RARE) in the promoter region of the retinoic acid-responsive genes to activate their expression [18,19,20].

## 3. Retinol and Pluripotency of Embryonic Stem (ES) Cells

In addition to its role in cell differentiation via retinoic acid, recent studies have demonstrated that vitamin A/retinol also has a direct function in the maintenance of self-renewal and prevention of differentiation of pluripotent stem cells [12,13,14,15]. These studies have demonstrated for the first time that vitamin A/retinol supports self-renewal of embryonic stem (ESCs) cells by elevating the expression of NANOG and OCT4, the critical transcription factors for the maintenance of pluripotency of ESCs [28]. Retinol executes its function by activating the phosphatidylinositol 3 (PI3) kinase signaling pathway via insulin like growth factor 1 (IGF1) receptor [12].

Mouse ESCs maintain their self-renewal in the presence of leukemia inhibitory factor (LIF) [29] but tend to differentiate spontaneously when LIF is removed from the medium. Other studies have shown that removal of LIF increases the metabolism of retinol into 4-hydroxyretinol and 4-oxoretinol by cytochrome P450 enzyme CYP26 (retinoic acid hydroxylase) with concomitant differentiation of ESCs without forming retinoic acid [30]. However, mouse ESCs treated with 0.2–0.5 μM retinol in the presence of LIF upregulated the expression of NANOG and OCT4 by 3–5 folds and prevented their differentiation [12].

Treatment of cells with LY294002, a potent inhibitor of PI3 kinase prevented the self-renewal of ESCs by retinol confirming the involvement of PI3 kinase signaling pathway [14]. The addition of inhibitors of various receptor tyrosine kinases such as AG1478, the inhibitor of epidermal growth factor receptor (EGFR) and picropodophyllin, the inhibitor of insulin like growth factor 1 receptor (IGF1R) revealed that picropodophyllin prevented the upregulation of NANOG and OCT4 by retinol proving that retinol signaling is mediated via IGF1R.

**Figure 2:**
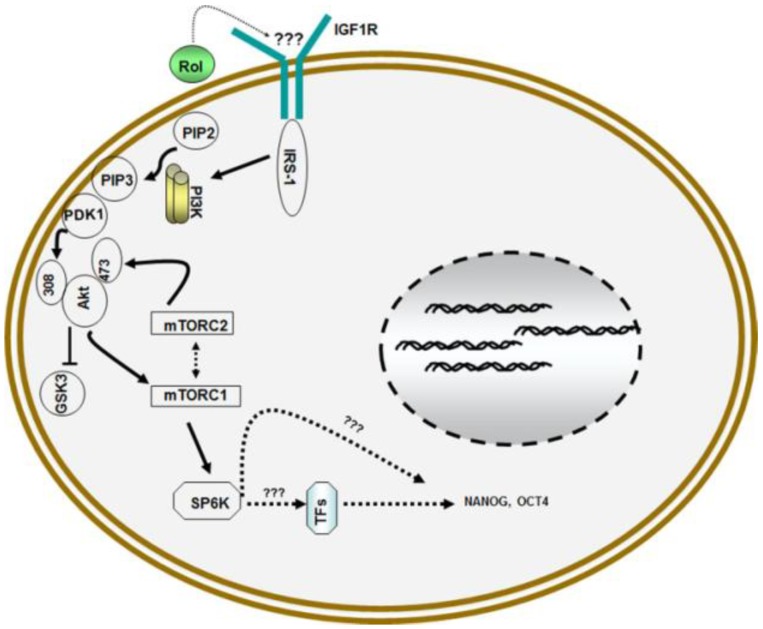
Model for retinol metabolism in ESCs: Vitamin A/retinol (Rol) activates PI3 kinase in ESCs by engaging IGF1R via yet to be determined mechanism that leads to the activation of Akt/PKB via phosphorylation at serine308 and serine473 which then activates downstream targets mTORCI and mTORC2 complexes which ultimately results in the activation of pluripotent stem cell specific gene NANOG and OCT4.

Further studies revealed that retinol signaling caused the activation of downstream effector of PI3 kinase PKB/Akt by enhanced phosphorylation at threonine308 and serine473. The activation of PKB/Akt then led to the regulation of mammalian target of rapamycin (mTOR) that controls protein synthesis by phosphorylating ribosomal protein p70S6 kinase (S6K) and eukaryotic initiation factor 4E (eIF4E) binding protein (4E-BP1) which is crucial for the synthesis of components of translation apparatus [31]. Both mTORC1 and mTORC2 complexes were activated by the retinol signaling [14] (Figure 2). However, the mechanisms by which vitamin A/retinol activates IGF1R and upregulation of NANOG and OCT4 via PI3 kinase signaling forms an interesting area for future explorations.

Contrary to its role in cell differentiation, Wang *et al*. [32] have revealed that short term exposure of murine ESCs to retinoic acid during early differentiation prevents spontaneous differentiation of these cells. The cells retain the capacity to differentiate into cardiomyocytes, neuronal cells and visceral endoderm, the derivatives of all three germ layers. Their studies also revealed that retinoic acid prevented the differentiation of ESCs by up regulating the expression of LIF, Wnt3a, Wnt5a, and Wnt6. Earlier studies by Miyabayashi *et al.* [33] on the other hand, have shown that Wnt/β-catenin/CBP signaling maintains the pluripotency of mouse ESCs.

Mouse and rat ESCs can self-renew in a defined medium containing fetal calf serum and LIF in which mitogen activated protein kinases (MAPK) signaling is eliminated and the activity of glycogen synthases (GSK3) activity is blocked by dual inhibitors (2i) using PD0325901 and CHIR99021 inhibitors [34]. Interestingly, retinol has no effect on GSK3 phosphorylation [11] or MAPK signaling (Chen and Khillan, unpublished results [35]).

## 4. Retinol and Proliferation of Germ Line Stem Cells (GSCs)

Recent studies by Zhang *et al*. [15] have shown that vitamin A/retinol also supports the self-renewal of mouse GSCs. Their studies revealed that mGSCs proliferated faster in retinol medium and exhibited strong proliferation and colonization. Further, RNA and protein analyses revealed the upregulation of genes involved in cell proliferation such as proliferating cell nuclear antigen (PCNA), c-Myc, cyclin D1, cyclin A and CDK2. PCNA is a marker for the proliferative spermatogonia [36,37] and cyclin A and cyclin D1 are involved in regulating the cell cycle progression from G1 to S phase. c-Myc activation on the other hand, regulates the cell proliferation, cell growth, apoptosis, differentiation and stem cell self-renewal.

The mGSCs cultured in retinol medium maintained pluripotency for over 18 passages as indicated by the high expression of NANOG, OCT4 and SOX2. Further, the mGSC colonies were also positive for cell surface markers of mouse ESCs such as SSEA1 and CD49f as well as *Vasa*, *Stella* and *Dazl* which are cell surface markers of mGSCs [38,39].

The pluripotency of mGSCs was further proven by teratoma formation after implantation into immunodeficient mice which contained derivatives of endodermal, mesodermal, and ectodermal embryonic germ layers including stratified cell epithelium, neuronal cells, cartilage, muscle, glandular structures, and endodermal high prismatic epithelium [15].

## 5. Retinol and Proliferation of Human ESCs

Treatment of human ESCs with vitamin A/retinol resulted in excellent morphology of the undifferentiated colonies, *i.e.*, even, thick, and big colonies. Also the number of undifferentiated colonies increased in the presence of retinol as accompanied by the decrease of differentiated colonies [40].

Vitamin A/retinol was further evaluated for its effect on the maintenance of undifferentiated human ESCs. Low concentrations such as 0.1–0.5 μM retinol had no effect on the self-renewal of human ESCs whereas 2.0 μM retinol enhanced their proliferation and induced the expression of human ESC specific markers. In the presence of 2.0 μM vitamin A/retinol, the colonies were noticeably larger as compared to control samples. Furthermore, vitamin A/retinol increased the expression of pluripotency-supporting genes, especially NANOG, which had a >20-fold relative expression level in the presence of 2.0–3.5 μM retinol. The effect of retinol was observed both at protein and mRNA expression of NANOG and OCT4 [40]. Flow cytometry analysis further confirmed the increase of stem cell markers TRA-160 and SSEA4 and revealed the increase in the number of cells expressing TRA-160 and SSEA4 by 43% and 63% respectively.

## 6. Retinol and Amplification of Cancer Stem Cells (CSCs)

CSCs comprise a rare population of cells in solid tumors that are believed to be responsible for tumor metastasis and relapse [41,42]. In spite of their discovery in acute mylogenous leukemia (AML) over fifteen years ago [43] and subsequent documented existence in many solid tumors including brain, lung, colon, prostate and breast tumors (Reviewed in [44]), the identity of CSC still remains questionable [45]. Tumor initiating cancer cells are generally isolated by cell sorting via flow cytometry using CD44^+^/CD24^low/−^, CD133^+^ and ESA^+^ cell surface markers or by aldehyde dehydrogenase 1 (ALDH1) using ALDEFLUOR florescent assay [44]. These markers however, are not exclusive to the CSCs and are expressed by non-CSCs as well [46,47] thus raising the doubts whether these cells represent “precursor stem cells” or the downstream progenitor cells.

Vitamin A/retinol on the other hand, maintained the growth of undifferentiated cancer like stem cells from mouse mammary tumors in long term culture while eliminating the non-CSCs [16]. The cells exhibited indefinite capacity for self-renewal, expression of OCT4 and NANOG as well as mammary stem cell specific markers CD29 (β1-integrin) and CD49f (α6-integrin) [48]. Pure populations of putative CSCs could also be isolated from human breast cancer cell lines including MCF7, MBA MD231 and SUM159 cells (Khillan and Sharma personal communication).

CSCs are believed to be resistant to conventional therapies such as radiation therapy and chemotherapy [49,50]. The cells isolated via retinol signaling therefore, showed resistance to radiation and chemo therapeutic agents and formed highly metastatic tumors after implantation into non-obese diabetic/severe combined immunodeficient (NOD/SCID) mice which is considered to be the gold standard test for CSCs. After differentiation, the cells expressed mammary specific markers such as β-casein and estrogen receptors [16].

## 7. Stem Cells and Impaired Retinol Metabolism

Analysis of ESCs revealed that these cells are unable to metabolize retinol into retinoic acid [14]. As shown in Figure 1, retinol is transported into the cytoplasm of the target cell through the cell surface receptor STRA6 [25] where it binds to CRBP followed by metabolization into active ligand retinoic acid. Analysis of normal and retinol treated ESCs revealed the absence of Adh4, Adh1 and RALDH2 enzymes [14]. Further, ESCs also did not show the expression of STRA6 which indicates that the cells do not contain the receptors to transport vitamin A/retinol into the cell. Further investigations revealed that the cells do not express CRBP as well [14] suggesting that retinol may have extracellular function in these cells. Interestingly, similar analysis of CSCs also showed the absence of retinol metabolizing enzymes [16]. The lack of retinol metabolizing machinery therefore, suggests that stem cells do not have the capacity to synthesize retinoic acid which is further supported by the observations that addition of retinoic acid leads to their complete differentiation [13]. However, MassSpec analysis may be necessary to rule this out completely.

The absence of expression of genes involved in vitamin A uptake from the blood may not be totally surprising as most cells that depend on vitamin A, do not take up vitamin A directly from the blood. Neurons in the adult brain for example which are not directly exposed to blood, depend on retinoic acid for regulating learning and memory as well as protein translation [51,52]. The cells that take up vitamin A are cells in the blood/brain barrier including choroid plexus. These cells express high levels of STRA6 receptor whereas neurons by themselves do not express genes involved in the vitamin A uptake. The photoreceptor cells in the eye that depend on vitamin A aldehyde as the chromophore to absorb light [53], are also not directly exposed to the blood due to the existence of blood/retina barriers. The vitamin A/retinol is therefore, absorbed by the RPE cells, which express STRA6 and take up a large amount of vitamin A/retinol. It is therefore, possible that the stem cells in stem cell niche which are not directly exposed to blood may not express the genes for vitamin A/retinol uptake. The vitamin A/retinol in these cells may become available either via diffusion or from neighboring cells that take up the vitamin from blood, with the exception for hematopoietic stem cells.

## 8. Purity of Stem Cell Population

Retinol treatment of mouse ESCs and induced pluripotent stem cells (iPS) cells resulted in the elimination of differentiated cells leaving only pure population of undifferentiated cells [54] suggesting that the differentiated cells may have been eliminated via terminal differentiation due to their ability to metabolize retinol into retinoic acid. The undifferentiated characteristics of these cells were confirmed by generating chimeric animals via injection of cells into mouse blastocysts. The microinjection of cells resulted in the chimeric animals that exhibited high percentage of ESC contribution accompanied by germ line transmission of coat color of the ESCs [54].

## 9. Retinol and Mitochondrial Function

Earlier, studies by Acin-Perez *et al*. [17] have revealed that vitamin A/retinol plays essential role in the metabolic fitness of the mitochondria. The cells deprived of vitamin A/retinol defaulted to basal levels of ATP synthesis which resulted in acute energy crisis. The cells recovered to significantly higher energy output as soon as the physiological level of retinol was restored without the need for conversion to other retinoids. c-Raf and protein kinase C (PKC) families of serine/threonine kinase [55] which plays critical role in mitochondrial biology, contain high affinity retinol binding sites in their regulatory domains [56]. PKCδ for example, contains high affinity binding sites for retinol in its zinc finger domain whereas, PKCδ/retinol complex signals the pyruvate dehydrogenase complex for enhanced flux of pyruvate into the Krebs cycle [17]. However, due to many isoforms, e.g., 11 for PKC and 3 for Raf as target molecules, the complexity of the problem has proven to be overwhelming to investigate the function of individual target. The role of vitamin A/retinol in controlling the mitochondrial oxidative phosphorylation in relation to stem cell biology presents an interesting area for future investigations.

## 10. Vitamin A and Reproduction

The need for dietary vitamin A/retinol for normal spermatogenesis has been recognized for decades [57,58,59,60]. Wolbach and Howe [57] first observed that vitamin A deficient rodents suffered blindness and infertility disorders. Rats that are fed vitamin A/retinol free diet but containing the retinoic acid grow well but become blind [61] and infertile [62] without the exhibition of other signs associated with vitamin A deficiency (VAD). Females rats on the other hand, maintained only on retinoic acid diet became pregnant but the fetuses were invariably resorbed suggesting that vitamin A alcohol has specific functions which cannot be compensated by retinoic acid alone.

Male rats maintained on only retinoic acid diet developed lesions in the reproductive tract. The rats also developed testicular changes such as sloughing of the cells of the germinal epithelium followed by an obliteration of the lumen of the tubule by Sertoli cells. However, the testicular regeneration was restored once the animals were fed diet containing vitamin A/retinol [58]. Withdrawal of retinol leads to arrest in the transition of A spermatogonia to A1 spermatogonia (reviewed in [63]). The normal spermatogenesis on the other hand, was restored when retinol was administered through diet [64,65] and the retinol treatment of VAD rats leads to synchronization of seminiferous tubules in the testis [66].

BMP4, a member of Bone morphogenetic proteins (BMPs) family has been implicated in spermatogenesis and germ cell survival [67]. Inactivation of BMP4 gene by gene targeting leads to the failure to form primordial germ cells (PGCs) [68]. It is also essential for PGC localization to the genital ridge and PGC survival [69]. BMP4 expression is significantly upregulated in testis of vitamin A deficient mice [70] which is down regulated by only retinol and not by retinoic acid. Further, germ cells utilize BMP4 intron 2 promoter in addition to classical 1A and 1B promoters. The decrease in BMP4 by retinol was found to be mediated by the 1A and intron 2 promoters [70]. Overall, the studies strongly support a direct role of vitamin A/retinol in reproduction. Further studies are needed to define this function at the molecular level.

## 11. Conclusions

Vitamin A/retinol is absorbed from food in the small intestine. It is stored in the liver from where it is mobilized into blood circulation bound to RBP4. Liver exhibits high affinity binding sites for RBP4 however, it does not express STRA6 receptor [25]. Recent studies have identified a new receptor RBP4-receptor2 (RBPR2) which is primarily expressed in liver and intestine and is induced in adipose tissue of obese mice. It is structurally related to STRA6 and is highly conserved in vertebrates [71]. This receptor is believed to regulate retinol homeostasis in these tissues. STRA6, on the other hand has been shown to catalyze efficient retinol exchange between intracellular CRBPI and extracellular RBP4. This retinol influx and efflux by STRA6 may serve to refresh the intracellular retinol pool [72].

Many reports now provide evidence for a retinoic acid independent direct function of retinol both *in vivo* [57,58,59,60,61,62,63,64,65,66,67] and *in vitro* [12,13,14,15,16,17]. In addition, the studies by See *et al*. [73] have shown that all the late vitamin A deficiency (VAD)-induced malformations in rat could be prevented by the addition of retinol starting at E10.5, whereas provision of all trans retinoic acid throughout pregnancy only led to the improvement but failed to completely rescue the development of all organ systems. Collectively, these studies provide strong evidence for direct and retinoic acid independent function of vitamin A/retinol. The mechanism/s by which retinol executes its function however, remain poorly understood mainly due to the complexity of the *in vivo* systems. The results obtained in *in vitro* studies have limitation for directly extrapolating to *in vivo* data as the retinol in cell culture media can readily distribute to the cell membrane. Incidentally, the stem cells do not express STRA6 receptor [14] and the retinol metabolizing enzymes [14,16]. The expression of RBPR2 that confers high affinity RBP4 binding and retinol transport [71] have not been reported on stem cells. Future studies will reveal how retinol interacts with stem cells to regulate cell proliferation and self-renewal.

Retinoids, the natural and synthetic analogues of retinol, have been of significant interest as cancer preventive agents since late 1970s [74,75,76]. They have been used in many clinical trials to assess the role of β-carotene in cancer prevention. Almost 27 years ago, the first phase of β-Carotene and Retinol Efficacy Trial (CARET) among men and women at high risk of lung cancer was stopped early primarily due to an increased risk of lung cancer [75]. Similarly α-tocopherol and β-carotene (ATBC) and CARET study found an increased risk of lung cancer among those assigned to active β-carotene treatment [77] or no significant benefit of β-carotene [78]. On the other hand, β-carotene alone was found to be associated with an increased risk of aggressive prostate cancer in a nested case-control study [79,80]. The outcome of these trials therefore, led some to question the safety of β-carotene supplementation for cancer prevention.

In the light of recent observations of retinol function in the self-renewal of stem cells [12,13,14,15] and CSCs [16], the unexpected outcome of the above trials arouses curiosity whether vitamin A/retinol may have enhanced the amplification of CSCs. In context with the outcome of cancer trials, the absence of retinol metabolizing machinery in ESCs that share many properties with cancer cells evokes a provocative hypothesis that CSCs may also lack the capability to metabolize retinol [16]. Interestingly, ESC like molecular phenotype is enriched in tumor compared to normal tissue and association between an ESC like phenotype and poor prognosis is observed in breast, lung, bladder and brain tumors [81,82]. Overall, the studies described in this review support the hypothesis that stem cells in general may not have the capacity to metabolize retinol to retinoic acid. This may have major implications for regenerative medicine for isolating stem cells from adult tissue as well as CSCs from solid tumors for the identification of biomarkers and developing strategies for management of cancer for personalized therapeutics. This review therefore, brings into sharp focus the new directions and challenges for the investigators in the field of retinoids that need to be explored to delineate the mechanisms by which the alcohol form of vitamin A influences the biological functions without conversion into its acid metabolite.
